# Test‐retest and time dependent variation and diagnostic values of vibratory sensation determined by biothesiometer and the Rydel‐Seiffer tuning fork

**DOI:** 10.1002/brb3.2230

**Published:** 2021-06-04

**Authors:** Bolette Wittenberg, Toke K. Svendsen, Laura M. Gaist, Mustapha Itani, Sandra S. Gylfadottir, Troels S. Jensen, David Gaist, Søren H. Sindrup, Thomas Krøigård

**Affiliations:** ^1^ Research Unit for Neurology Odense University Hospital University of Southern Denmark Odense Denmark; ^2^ Danish Pain Research Center Department of Clinical Medicine Aarhus University Aarhus Denmark; ^3^ Department of Neurology Aarhus University Hospital Aarhus Denmark

**Keywords:** biothesiometer, polyneuropathy, Rydel‐Seiffer, tuning fork, vibration detection threshold

## Abstract

**Background and aims:**

Polyneuropathy is a common neurological disorder with many potential causes. An essential part in screening, diagnosis, and follow‐up evaluation of polyneuropathy is testing of the sensory function including vibratory sensation. The graduated Rydel‐Seiffer tuning fork and the biothesiometer have been developed to quantify vibratory sensation through detection thresholds. The aim of this study is to compare the vibration detection thresholds determined by the two instruments regarding intraindividual temporal changes, interindividual variation in healthy subjects and comparison of the diagnostic value in patients with a clinical suspicion of polyneuropathy.

**Methods:**

Ninety‐four healthy subjects, 98 patients with and 97 patients without a diagnosis of polyneuropathy were included. Quantitative sensory testing including biothesiometry, structured clinical examination, and nerve conduction studies were performed three times during 52 weeks in healthy subjects and once in patients.

**Results:**

There were no significant changes over time for neither the Rydel‐Seiffer tuning fork nor the biothesiometer, and both had larger between‐subject variation than within‐subject variation. Relative intertrial variability was largest for the biothesiometer. Diagnostic value (sensitivity, specificity, positive predictive value, and negative predictive value) was moderate for both methods (Rydel‐Seiffer tuning fork: 58%, 74%, 70%, 64%; biothesiometer: 47%, 77%, 68%, 59%).

**Interpretation:**

The Rydel‐Seiffer tuning fork and the biothesiometer have a low test‐retest and time dependent variation. They perform almost equally as diagnostic tools in patients with suspected polyneuropathy with a tendency toward better performance of the tuning fork.

## Graphical abstract

The graduated Rydel‐Seiffer tuning fork and the biothesiometer have been developed to quantify vibratory sensation through detection thresholds. We compared intra‐individual temporal changes, inter‐individual variation in healthy subjects and comparison of diagnostic value in patients with a clinical suspicion of polyneuropathy. They both have a low test‐retest and time dependent variation and perform almost equally as diagnostic tools in patients with suspected polyneuropathy with a tendency towards better performance of the tuning fork.

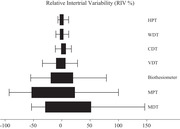

## 1 INTRODUCTION

Polyneuropathy is a common neurological disorder. The overall prevalence ranges from 1% to 3%, and in the elderly, it increases to 7% (Hanewinckel et al., 2016). Many potential causes and risk factors have been identified with diabetes and alcohol overuse being two of the most common factors related to polyneuropathy (Hanewinckel et al., 2016). An essential part of the screening, diagnosis and follow‐up evaluation of polyneuropathy is the assessment of sensory function, including vibratory sensation. Vibration and pinprick sensation along with ankle reflexes were found to be the most sensitive measures of polyneuropathy in clinical testing (Abraham et al., 2017), whereas both ankle reflexes and vibratory sensation had the lowest specificities as they were often absent or reduced in elderly.

Vibratory sensation is usually tested using a simple 128 Hz tuning fork during standard neurological examination. As a consequence of the central role of vibratory sensation in evaluation of polyneuropathy, quantitative methods have been developed to determine vibration detection thresholds (VDT), including the Rydel‐Seiffer tuning fork and the biothesiometer.

The Rydel‐Seiffer tuning fork reports VDT on a scale from 0 (vibration sense absent) to 8, and testing has been standardized as a part of quantitative sensory testing (QST) described by the German Research Network on Neuropathic Pain (Rolke, Baron, et al., 2006; Rolke, Magrel, et al., 2006). The graduated tuning fork is a simple and rapid method to assess vibratory sensation (Thivolet et al., 1990).

On the other hand, the biothesiometer (Bloom et al., 1984) determines VDT on a scale from 0 to 50 by adjusting the amplitude of an electrical vibrator, which provides a quantitative measure of vibratory sensation (Young et al., 1993). Assessment of VDT using the biothesiometer is quick and reliable (Bloom et al., 1984), and in comparison with a standard tuning fork the biothesiometer is reported to be more accurate (Temlett, 2009).

The aim of this study was to compare VDTs determined using the graduated Rydel‐Seiffer tuning fork and the biothesiometer. In healthy subjects, the specific aims of the study were (1) to determine the intraindividual changes over time and (2) to determine the interindividual variation and in patients with a clinical suspicion of polyneuropathy (3) to compare the diagnostic value (sensitivity, specificity, positive predictive value (PPV), and negative predictive value (NPV)).

## 2 SUBJECTS AND METHODS

### 2.1 Repeated measurements and reference values

Two groups of healthy subjects were used to determine reference values for QST: Group 1 consisted of 46 healthy subjects recruited (1) from the local community via advertisements and (2) among spouses to patients examined in a previous study (de Koning Svendsen et al., 2020). The subjects were included if they were aged 40 to 80 years and had no symptoms or signs of polyneuropathy. Blood samples at baseline were analyzed in order to exclude subjects with underlying diseases. Exclusion criteria have previously been described (de Koning Svendsen et al., 2020), and included, among others, polyneuropathy symptoms, known risk factors for polyneuropathy before entering the study, or prior treatment with statin or other cholesterol‐lowering drugs before entering the study. QST and biothesiometry were performed three times during the study period: at inclusion (baseline), after 6 weeks and after 52 weeks. QST and biothesiometry were performed by two study physicians certified in QST according to the German Research Network on Neuropathic Pain at the Department of Neurophysiology in Mannheim. Data from these healthy subjects were also used to determine temporal variability.

Group 2 consisted of 50 healthy subjects recruited (1) among staff at Odense University Hospital and (2) if they were in a social or work circle of patients with diabetes that participated in a previous study (Gylfadottir et al., 2020). These healthy subjects had served as a control group in the previous study. They were excluded if they had diabetes, severe chronic illness, psychiatric or neurologic illness, chronic pain or had been taking pain medication three days before entering the study. QST and biothesiometry were performed once, however testing did not include heat pain threshold (HPT) or mechanical pain threshold (MPT). QST and biothesiometry were performed by two study nurses certified in QST according to the German Research Network on Neuropathic Pain at the Department of Neurophysiology in Mannheim.

### 2.2 Performance in target population (diagnostic values)

Patients with a diagnosis of polyneuropathy and patients without a diagnosis of polyneuropathy were identified from a database at the neuromuscular clinic at Odense University Hospital. The database, from which data was extracted, contains comprehensive clinical data regarding neuropathy symptoms and signs from a large group of patients examined due to a clinical suspicion of polyneuropathy since 2016. The diagnosis of polyneuropathy was made by experienced neuromuscular neurologists based on both clinical information and extensive small and large fiber neuropathy diagnostic work‐up, including among other nerve conduction studies, skin biopsies, QST, and corneal confocal microscopy. QST did not include HPT.

The diagnosis of polyneuropathy was based on either (1) typical symptoms and signs of polyneuropathy and abnormal nerve conduction study (NCS) or skin biopsies as described below or (2) typical symptoms and signs of polyneuropathy and relevant additional diagnostic work up to exclude alternative diagnoses. Specifically, lumbar MRI was performed to exclude lumbar root compression in patients with NCS, which did not fulfill the criteria for polyneuropathy.

Neuropathy subtype was determined based on nerve conduction studies, skin biopsies, thermal, vibration and mechanical detection thresholds, and clinical examination as previously described (model 1) (Itani et al., 2021).

Both patients with and without a diagnosis of polyneuropathy were matched for sex and age to the healthy controls. For each of the healthy subjects, the patient with and the patient without polyneuropathy matching most closely regarding age to the healthy subject was included. If possible (both male and female patients with the same age), sex was also matched. Due to the large size of the data base matching for age ± 3 years was possible in all cases.

### 2.3 Quantitative sensory testing

QST was performed according to the standards of the German Research Network on Neuropathic Pain (DFNS) (Rolke, Baron, et al., 2006, Rolke, Magrel, et al., 2006 ). Testing was performed on the dorsum of the right foot, while tests of vibratory sensation were performed at the tip of the right first toe. All results were determined as the mean of three tests.

QST included determination of VDT using a Rydel‐Seiffer graduated tuning fork (64 Hz, scale 0–8 units), mechanical detection threshold (MDT) using von Frey filaments, MPT using pinprick stimuli and warmth, cold and heat pain detection thresholds (WDT, CDT, HPT) using a TSA II‐NeuroSensory Analyzer (Medoc, Israel). The biothesiometer (Bio‐medical Instruments CO, Newbury, Ohio, USA), which is not part of standard QST, was also used to determine vibration sensitivity. The biothesiometer (50 Hz, scale 0–50 volt) was hand‐held and resting with its own weight with the probe vibrating at the tip of the first toe. The voltage was slowly increased until the subject felt the vibration for the first time and the corresponding voltage was recorded. The results of the standard assessment of vibratory sensation (felt/not felt at the first toe) using a nongraduated 128 Hz tuning fork was extracted from the database.

### 2.4 Nerve conduction studies, skin biopsies, and corneal confocal microscopy

Nerve conduction studies were performed using standard surface electrodes. Sural, tibial, and peroneal nerves were examined bilaterally and median and ulnar nerves were examined unilaterally. Nerve conduction studies were considered abnormal if at least two nerves had at least one abnormal variable, one of which must be the sural nerve.

Skin biopsies from 10 cm proximal to the right lateral malleolus were processed according to European Federation of Neurological Societies (EFNS) and the Peripheral Nerve Society (PNS) guidelines (Lauria et al., 2005) and intraepidermal nerve fiber density was compared to a large international reference material (Lauria et al., 2010).

Corneal confocal microscopy was performed as previously described (Tavakoli & Malik, 2011), and considered abnormal in case of reduced nerve fiber density or nerve fiber length.

Nerve conduction studies and skin biopsies were performed in both healthy controls and patients. Corneal confocal microscopy was only performed in patients.

### 2.5 Ethics

The data retrieved from both healthy subjects and patients for this study were approved by the Ethics Committee of the Region of Southern Denmark and the Danish National Committee on Health Research Ethics (Project ID: S‐20140089, S‐20100082 and S‐20150166) and registered at the Danish Data Protection Agency (Project ID: 14/47400, 2008‐58‐0035 and 15/51881).

### 2.6 Statistical analyses

Data from the healthy subjects with repeated measurements was analyzed in order to determine intra‐individual changes over time and interindividual variation using a generalized linear mixed model (random‐effects model). Within‐subject (random effects) and between‐subject variations were determined with time, age, and sex as fixed effects. The intraclass correlation coefficient (ICC) was calculated as the between‐subject variance divided with the total variance. Changes per week (95% confidence intervals) was also calculated. Bland‐Altman plots were constructed. Furthermore, the relative intertrial variability (RIV) was calculated as the difference between the first and the third examination divided by the first examination.

For the analysis of diagnostic values, cut‐off values at 5th or 95th percentiles depending on the specific QST method were determined. Sensitivity, specificity, PPV, and NPV for the final diagnosis of polyneuropathy, based on clinical information and the diagnostic work‐up described above, were calculated based on these cut‐off reference values.

All analyses were performed using STATA IC version 16 including the extension www.gllamm.org (random‐effects model calculations).

## 3 RESULTS

For the analysis of temporal changes and variation, full data sets were available for 37 subjects. Seven subjects had missing values for either “6 weeks examination” or “52 weeks examination.” Two subjects had signs of subclinical polyneuropathy on clinical examination (reduced vibration sensitivity) and they were excluded from all analyses. The population of healthy subjects for reference values was 94.

We identified 195 patients, including 98 patients with and 97 patients without a diagnosis of polyneuropathy from the database.

Characteristics of healthy subjects and patients are listed in Table 1. Median age was comparable, and male sex varied between 34% and 36%. As described above, the healthy subjects composed of two groups; the first group (*n* = 44) had a median age of 50.0 years (range 40–80) and 36% were men, whereas the second group of healthy subjects (*n* = 50) had a median age of 63.0 years (range 28–79) and 32% were men.

**TABLE 1:** Patient characteristics

	+PNP(*n* = 98)	−PNP(*n* = 97)	Healthy subjects(*n* = 94)
Age, median (range), years	55 (29−81)	55 (27−81)	55 (28−80)
Sex, male (%)	35 (35.7)	35 (36.1)	32 (34.0)
NIS total, median (range)	10 (0−50)	−	−
NPSI total, median (range)	24 (0−88)	−	
Diagnostic tests, abnormal (%)			
Nerve conduction studies	34 (35)	0 (0)	
Skin biopsy	42 (43)	2 (2)	
Corneal confocal microscopy	30 (31)	9 (9)	
Neuropathy subtype, patients (%)			
Mixed fiber	56 (57)		
Large fiber	32 (33)		
Small fiber	10 (10)		
Etiology			
Diabetes	18		
Alcohol	10		
Diabetes and alcohol	3		
Chemotherapy	11		
Other medication	5		
Connective tissue disease	5		
Thyroid disease	3		
MGUS	2		
Sarcoidosis	2		
Malnutrition	1		
Other	2		
Unknown	36		
Alternative diagnosis			
Lumbar root compression		13	
Medication side effect		4	
Edema		2	
Unspecific chronic pain		2	
Erythromelalgia		1	
Sarcoidosis		1	
Functional disorder		1	
Cobalamin deficiency		1	
Unknown		72	

Abbreviations: NIS, neuropathy impairment score; NPSI, neuropathic pain symptom inventory; +PNP, patients with a polyneuropathy diagnosis.; −PNP, patients without a polyneuropathy diagnosis.

There were no significant changes over time for neither the Rydel‐Seiffer tuning fork (VDT) nor the biothesiometer, whereas this was the case for CDT and MPT (Table 2).

**TABLE 2:** Repeated quantative sensory testing in healthy subjects

	Baseline, Mean (SD)	6 weeks, Mean (SD)	52 weeks, Mean (SD)	Change per week (95 % CI)	*p*	Within‐subject variation, SD	Between‐subject variation, SD	ICC
VDT, R‐S	6.5 (1.1)	6.0 (1.4)	6.3 (1.3)	0.0004 (−0.005; 0.006)	.887	0.68	0.82	0.60
Biothesiometer	10.2 (4.7)	10.6 (5.1)	9.9 (4.6)	0.0095 (−0.029; 0.0096)	.33	2.39	3.2	0.64
CDT (°C)	27.2 (3.7)	27.2 (5.0)	28.1 (3.5)	0.018 (0.001; 0.034)	.032	2.02	2.75	0.65
WDT (°C)	38.9 (3.6)	38.0 (3.0)	38.9 (3.5)	0.008 (−0.008; 0.023)	.331	1.91	2.12	0.55
HPT (°C)	46.2 (2.8)	45.8 (2.7)	46.4 (2.5)	0.008 (−0.008; 0.024)	.311	1.98	1.34	0.31
MDT (mN)	5.3 (4.0)	5.4 (3.3)	6.7 (9.1)	0.028 (−0.012; 0.069)	.175	5.06	2.59	0.21
MPT (mN)	42.9 (32.3)	39.6 (37.6)	32.5 (28.6)	−0.183 (−0.333; −0.033)	.017	18.72	22.11	0.58

Abbreviations: CDT, cold detection threshold; HPT, heat pain threshold; ICC, intraclass correlation coefficient; MDT, mechanical detection threshold; MPT, mechanical pain threshold; R‐S, Rydel‐Seiffer; SD, standard deviation; VDT, vibration detection threshold; WDT, warmth detection threshold.

HPT and MDT were the only QST methods with a larger within‐subject variation compared to between‐subject variation, whereas the other QST methods, including Rydel‐Seiffer VDT and the biothesiometer, had a larger between‐subject variation. ICC ranged from 0.21 to 0.65. Rydel‐Seiffer VDT and the biothesiometer had ICC values of 0.60 and 0.64, respectively, whereas MDT had the lowest ICC followed by HPT. CDT had the highest ICC (Table 2).

**FIGURE 1:**
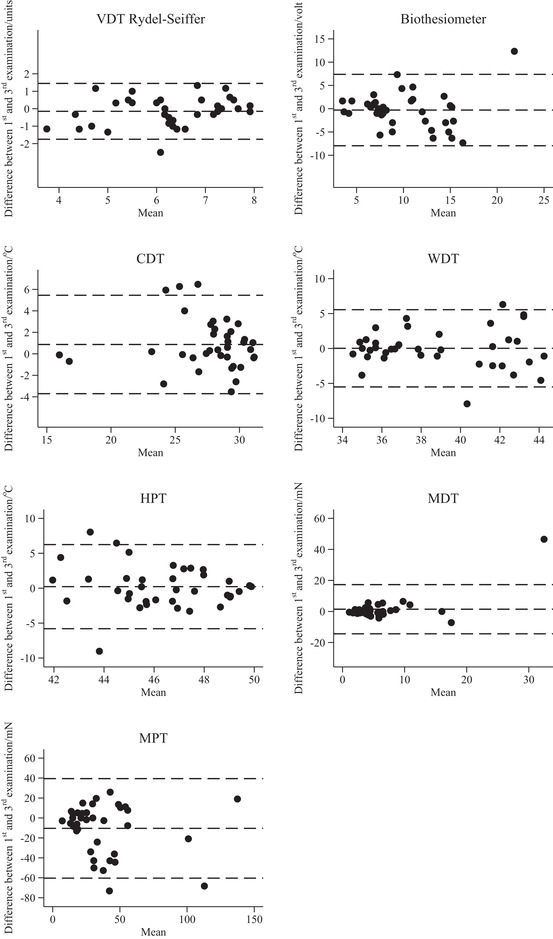
Bland‐Altman plots for all quantitative sensory testing methods. Each dot represents a healthy subject (*n* = 37). Dotted lines represent mean and mean ± 1.96 SD. CDT: Cold detection threshold. HPT: Heat pain threshold. MDT: Mechanical detection threshold. MPT: Mechanical pain threshold. VDT: Vibration detection threshold. WDT: Warmth detection threshold

**FIGURE 2:**
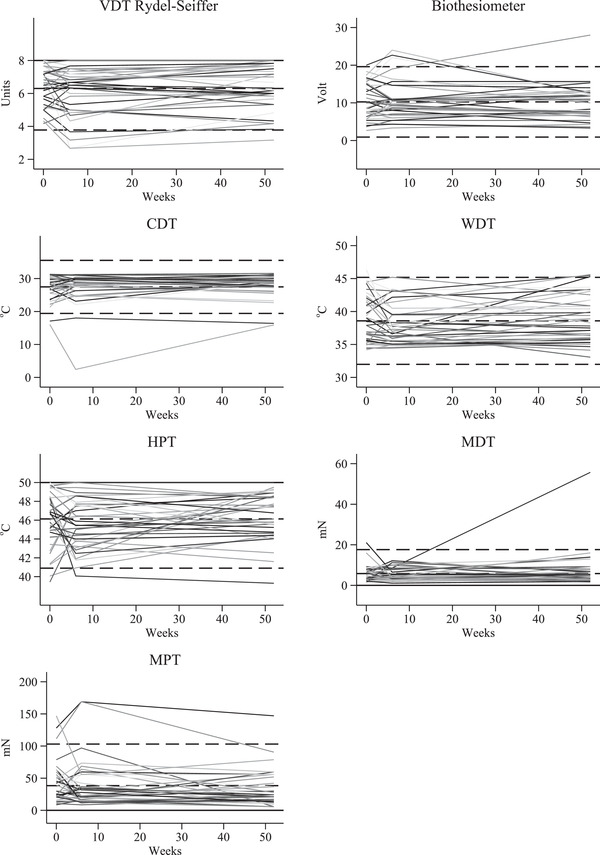
Repeated examination of healthy subject (baseline, 6 weeks and 52 weeks) for all quantitative sensory testing methods. Each line represents a healthy subject (*n* = 37) at three examinations. Dotted lines represent mean and mean ± 1.96 SD. CDT: Cold detection threshold. HPT: Heat pain threshold. MDT: Mechanical detection threshold. MPT: Mechanical pain threshold. VDT: Vibration detection threshold. WDT: Warmth detection threshold

The Bland‐Altman plots (Figure 1) show that Rydel‐Seiffer VDT and biothesiometer measurements were within 95% limits of the normal distribution (mean ± 1.96 SD) except from one subject for both measures. In general, change between the first and third examination was independent of the mean of the two examinations. Furthermore, analyzing individual changes during the study period (Figure 2), for both Rydel‐Seiffer VDT and the biothesiometer change in status from within to beyond the 95% limits between baseline and 52 weeks examination was very rare (one subject for each measure).

**FIGURE 3:**
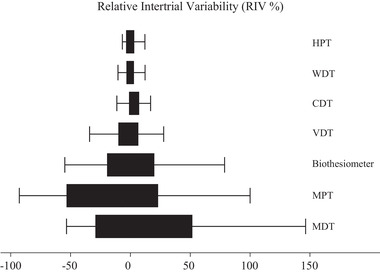
Relative intertrial variability (RIV) for all quantitative sensory testing methods from repeated examination of healthy subjects (*n* = 37). Boxes indicate 25th to 75th percentile. 11 outliers were omitted (CDT 3, HPT 3, WDT 2, MDT 2and biothesiometer 1). CDT: Cold detection threshold. HPT: Heat pain threshold. MDT: Mechanical detection threshold. MPT: Mechanical pain threshold. VDT: Vibration detection threshold. R‐S: Rydel‐Seiffer. WDT: Warmth detection threshold

Thermal sensory thresholds (HPT, WDT, and CDT) had a low relative intertrial variability (RIV) compared to other measures, including VDT and the biothesiometer. HPT had the lowest RIV and MDT and MPT had the largest RIV (Figure 3). The 25th–75th percentiles for Rydel‐Seiffer VDT and the biothesiometer were −10.0% to 6.6% and −19.5% to 20%, respectively.

The 5th percentile cut‐off for Rydel‐Seiffer VDT was 3.83 units and the 95th percentile cut‐off of the biothesiometer was 27.3 volts.

Diagnostic values for Rydel‐Seiffer VDT, the biothesiometer, CDT, WDT, and MDT are presented in Table 3. Diagnostic value of the standard nongraduated tuning fork used in structured neurological examination is included for comparison. Rydel‐Seiffer VDT had the highest PPV (70%) and NPV (64%), whereas the nongraduated tuning fork and CDT had the highest sensitivity (61%) and specificity (86%), respectively. The biothesiometer had diagnostic values lower than VDT except from the specificity, which was 77%.

**TABLE 3:** Diagnostic sensory tests in patients with polyneuropathy

Sensory modality	Sensory test	Sensitivity (%)	Specificity (%)	PPV (%)	NPV (%)
Vibration	VDT, R‐S	58	74	70	64
	Biothesiometer	47	77	68	59
	Non‐graduated tuning fork	61	63	63	61
Mechanical	MDT	41	74	62	55
Thermal	CDT	29	86	67	54
	WDT	40	81	68	57

Abbreviations: CDT, cold detection threshold; MDT, mechanical detection threshold; NPV, negative predictive value; PPV, positive predictive value; R‐S, Rydel‐Seiffer; VDT, vibration detection threshold; WDT, warmth detection threshold.

**FIGURE 4:**
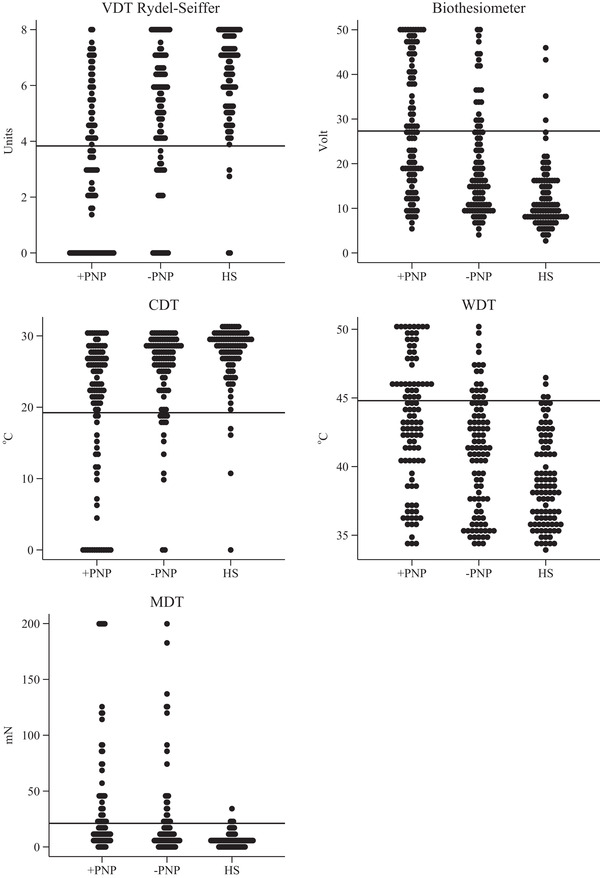
Patients with and without a diagnosis of polyneuropathy and healthy subjects (*n* = 98, 97, 94). Solid lines represent the reference value (5th or 95th percentile dependent on the specific quantitative sensory testing method). CDT: Cold detection threshold. HS: Healthy subject. MDT: Mechanical detection threshold. MDT. VDT: Vibration detection threshold. WDT: Warmth detection threshold. +PNP: Patients with a polyneuropathy diagnosis. −PNP: Patients without a polyneuropathy diagnosis

The distribution of measures in healthy subjects and patients with and without a diagnosis of polyneuropathy and the relation to cut‐off values based on 5th and 95th percentiles of the healthy subjects are presented in Figure 4.

## 4 DISCUSSION

The main findings of the study were that vibration detection thresholds determined by both the Rydel‐Seiffer tuning fork and the biothesiometer were stable and did not change significantly over time. The methods performed quite similarly regarding reproducibility, but relative intertrial variability was lower for the tuning fork. The instruments performed almost equally as diagnostic tools in patients with suspected polyneuropathy. Both the Rydel‐Seiffer tuning fork and the biothesiometer had a higher specificity and a lower sensitivity than a nongraduated tuning fork.

We found no significant temporal changes in VDT for neither the Rydel‐Seiffer tuning fork nor the biothesiometer. Both methods had larger between‐subject variation than within‐subject variation and both methods had intraclass correlation coefficients larger than 0.5. Our results are comparable to those of a previous study (Nothnagel et al., 2017), which analyzed the long‐term test‐retest reliability of QST. In that study, during a 10‐week study period, measurements obtained using the Rydel‐Seiffer tuning fork did not change over time and a similar ICC of 0.62 was reported.

It is reassuring that test‐retest variation is independent of the mean of the observations as illustrated by Bland‐Altman plots, a finding which supports that VDTs will be suitable for clinical and research follow‐up studies.

We found that thermal thresholds had the lowest relative intertrial variability followed by the Rydel‐Seiffer tuning fork and the biothesiometer. Compared to MDT, which is another test of large fiber function, both the Rydel‐Seiffer tuning fork and the biothesiometer had lower relative intertrial variability. The reason for these differences is unknown.

CDT and MPT changed significantly over time and changes in some QST variables were also reported by Nothnagel et al. (2017). As changes occurred between 6 and 52 weeks examinations, it seems unlikely that they are caused by habituation of study participants to study conditions, and they probably occurred by chance.

The Rydel‐Seiffer tuning fork had better diagnostic performance than the biothesiometer except for specificity, but for both methods values were only moderate. Other studies have previously compared the Rydel‐Seiffer tuning fork with an electrical device (e.g., the Vibrameter) in a clinical setting (de Simone et al., 2015, Martina et al., 1998). The Rydel‐Seiffer and the Vibrameter had sensitivities of 76% and 73%, respectively, for detection of mild polyneuropathy (Martina et al., 1998), and there was a moderately significant correlation between vibration thresholds. Sensitivities were higher than in the present study but the comparison is limited by differences in reference values which were age dependent in the previous study. Furthermore, the electrical instruments (biothesiometer and vibrameter) were different. In diabetic polyneuropathy (de Simone et al., 2015), the Rydel‐Seiffer tuning fork had a higher sensitivity and a lower specificity than the biothesiometer, which was also the case in our study.

The biothesiometer has previously been compared with nerve conducting studies and quantitative tests of thermal sensation for detection of large and small fiber neuropathies (Sindrup et al., 2001). Diagnostic performance of the biothesiometer when testing on thumb and first toe was found to be slightly better than in our study, except for NPV. As in the present study, sensitivity was low (56%). In this previous study, addition of biothesiometer measurements to nerve conduction studies did not significantly change the diagnostic sensitivity, which was the case for small fiber QST measurements.

For comparison (Tankisi et al., 2019), the sensitivity of nerve conduction studies for the diagnosis of polyneuropathy does not exceed 75% when the diagnostic criterion is at least one abnormal parameter detected (when testing a specific nerve) and when at least two abnormal nerves were required, sensitivity is even lower. The sural nerve examined using surface electrodes had a low sensitivity of 49%.

Among the QST methods, the vibration tests had the best sensitivity. We found that the nongraduated tuning fork used in standardized neurological examinations was more sensitive than the Rydel‐Seiffer tuning fork, which was surprising. However, a previous study comparing the use of a standard tuning fork against quantitative vibration thresholds (Burns et al., 2002) found that examiners overestimated loss of vibratory sensation when using a nongraduated tuning fork, which is also suggested by the low specificity of the nongraduated tuning fork in the present study.

The most important limitation of this study is interpretation of diagnostic values based on the use of our own reference values, which were not age adjusted. Patients and heathy subjects were matched for age and sex to minimize bias, but as shown in previous studies (Bloom et al., 1984, Rolke, Magrel, et al., 2006) age has an influence on VDT and diagnostic performance must be interpreted with caution. However, we believe that this does not affect conclusions regarding the primary aim of the study, which is comparison of Rydel‐Seiffer tuning fork and the biothesiometer. Another limitation is that the diagnosis of polyneuropathy was based on both clinical information and the results of diagnostic tests including QST. Therefore, there is a potential for circular reasoning regarding the diagnostic values of sensory testing.

The results have implications for evaluation of vibratory sensation in clinical practice and research. First, sensitivity for the diagnosis of polyneuropathy of both the Rydel‐Seiffer tuning fork and the biothesiometer did not exceed the sensitivity of the nongraduated tuning fork. This finding indicates that neither of these instruments for determination of VDT offers any advantage over the nongraduated tuning fork as a screening tool to support the clinical suspicion of polyneuropathy. On the other hand, specificity was substantially higher for both instruments, indicating that addition of these tests to clinical practice will improve the bedside ability to identify patients with alternative diagnoses. For research purposes, both instruments provided stable measurements. The Rydel‐Seiffer tuning fork had a lower relative intertrial variability than the biothesiometer. The biothesiometer does not seem to offer any advantage over the pocket‐sized Rydel‐Seiffer tuning fork regarding reproducibility, and further the instrument is less portable, dependent on electricity and more expensive.

In conclusion, for clinical and research purposes, the biothesiometer does not seem to offer any advantage over the more easily used Rydel‐Seiffer tuning fork.

## CONFLICT OF INTEREST

The authors have no conflict of interest to disclose.

### PEER REVIEW

The peer review history for this article is available at https://publons.com/publon/10.1002/brb3.2230.
